# A petal breakstrength meter for Arabidopsis abscission studies

**DOI:** 10.1186/1746-4811-2-2

**Published:** 2006-02-16

**Authors:** Kevin A Lease, Sung Ki Cho, John C Walker

**Affiliations:** 1 Division of Biological Sciences, 303 Life Sciences Center, University of Missouri, Columbia, MO 65211, USA

## Abstract

**Background:**

Abscission is the regulated dropping of plant organs, such as leaves or flower petals. This process involves a break down of the cell wall between layers of cells in the abscission zone, causing the organ to become detached. The model plant *Arabidopsis thaliana *undergoes floral organ abscission. Various experimental methods have been used to study Arabidopsis floral organ abscission, including measuring the petal breakstrength, or the amount of force required to pull a petal from the receptacle. Petal breakstrength provides a quantitative insight into the physical integrity of the petal abscission zone.

**Results:**

We developed a petal breakstrength meter that allows rapid data acquisition on a personal computer. We present the design of the device and show its utility in measuring Arabidopsis petal breakstrength for abscission studies.

**Conclusion:**

This petal breakstrength meter should enable researchers to perform the petal breakstrength assay as a routine part of the characterization of environmental and genetic factors affecting abscission.

## Background

The phenomenon of trees dropping their leaves in autumn is an example of abscission familiar to most people. Abscission, the regulated shedding of plant organs, can involve loss of other plant organs besides leaves, such as flower petals. At the cellular level, abscission involves a separation between layers of cells in the abscission zone, allowing a plant organ to detach [1,2]. For the separation to occur in a controlled fashion, there must be regulated expression of the enzymes involved in breaking down the components of the cell wall in the middle lamella.

A powerful method for identifying the regulatory components of abscission has been the study of *Arabidopsis thaliana *mutants that affect this process. Unlike many plants, Arabidopsis does not shed its leaves. However, Arabidopsis does undergo floral organ abscission, such as petal dropping. By identifying mutants with altered abscission, gene products and pathways important in abscission have been uncovered. For example, ethylene perception has been shown to be important in this response [3]. Furthermore, ACTIN-RELATED PROTEIN7 (ARP7) knockdowns exhibited delayed floral abscission [4]. Moreover, a cell surface receptor involved in abscission is HAESA, a leucine-rich repeat receptor kinase [5]. Inflorescence Deficient in Abscission (IDA), encodes a small secreted protein that is necessary for abscission [6]. BLADE-ON-PETIOLE1 and BLADE-ON-PETIOLE2 genes affect floral organ abscission [7]. Overexpression of AGL15 causes abscission defects (Fernandez et al., 2000). Aditionally, AUXIN RESPONSE FACTOR1 and AUXIN RESPONSE FACTOR2 mutants also affect abscission [8,9].

To understand the mechanistic basis of the abscission defects in these Arabidopsis mutants, several experimental approaches have been used. For example, the abscission zone has been visualized by microscopy [6]. In addition, the expression of a molecular reporter associated with abscission zones has been investigated [3]. Another assay that has been used in a subset of Arabidopsis abscission studies is the petal breakstrength assay [10,6,3]. In this assay, the force required to pull a petal from the receptacle is measured. This assay gives quantitative insight into the physical integrity of the abscission zone. For example, if the abscission zone is defective, more force may be required to pull the petal off than if abscission has been activated.

Part of the reason this assay has only been employed in a subset of Arabidopsis abscission studies may be that the device required to perform the assay is not a common piece of laboratory equipment. Furthermore, there is no off-the-shelf product that can be purchased to perform the assay, instead it must be built by the investigator. A petal breakstrength meter has been briefly described by Patterson and Bleecker (2004), which consisted of a FORT10 stress transducer (World Precision Instruments, Sarasota, FL) and a voltmeter. Further details of the device have only been published in a doctoral dissertation chapter [11], and so are not widely available. When we sought to build a petal breakstrength meter, we initially sought to purchase the FORT10 transducer, but we found that the device was no longer in production and was unavailable. Therefore, we designed the petal breakstrength meter around another force sensor which is in production, the MLT050 (ADInstruments, Colorado Springs, CO). We gave the petal breakstrength meter an additional feature: high speed data acquisition and logging to a personal computer. The design and use of our petal breakstrength meter should enable other researchers to build the meter for their abscission studies.

## Results

### Basic operation of the device in the petal breakstrength assay

**Figure 1:**
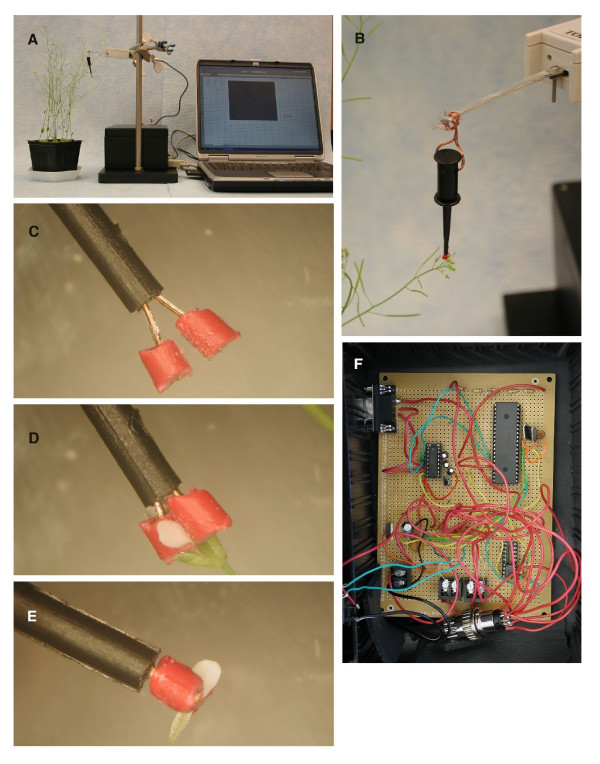
Petal breakstrength meter overview. **(A) **The petal breakstrength meter consists of a petal gripper, a sensor, electronic circuit and personal computer. **(B) **Close up of sensor showing 5 cm aluminum bar attached to sensor cantilever. Petal gripper is attached to the distal end of lever with a piece of braided wire. **(C-E) **Petal gripper (SMD grabber with 1 mm pieces of 20 gauge insulation glued to tips) in use. **(C) **Petal gripper is opened. **(D) **Grabbing onto a flower petal that is still attached to flower. **(E) **After pulling on the inflorescence, petal detaches from receptacle and remains in the jaws of petal gripper. **(F) **Assembled electronic circuit housed in an ABS plastic enclosure (lid removed).

The petal breakstrength meter consists of a petal gripper, a force sensor, an electronic circuit and a personal computer (Fig. 1A). The petal gripper hangs from the end of a 5 cm aluminum strip attached to the force transducer (Fig. 1B). The user first pushes down on the spring loaded plunger of the petal gripper to spread the gripper tips apart (Fig. 1C) Next, the gripper tips are positioned around the flower petal and the plunger is released, allowing the tips to move in and squeeze the petal (Fig. 1D). Data is acquired through the serial port of a PC using the Windows XP operating system, running the HyperTerminal application included in Windows. The COM port associated with the meter is selected and the following communication settings are selected: baud rate is 9600, 8 bits of data, 1 stop bit, no data flow. The device acquires over 100 voltage measurements per second, giving high temporal measurement resolution during the assay. Data is captured and saved to a file on the computer using the text capture feature of HyperTerminal. Text capture is started, the inflorescence is pulled down gently until the petal is pulled out of the flower (Fig. 1E) and then text capture is stopped.

### Explanation of the circuit

**Figure 2:**
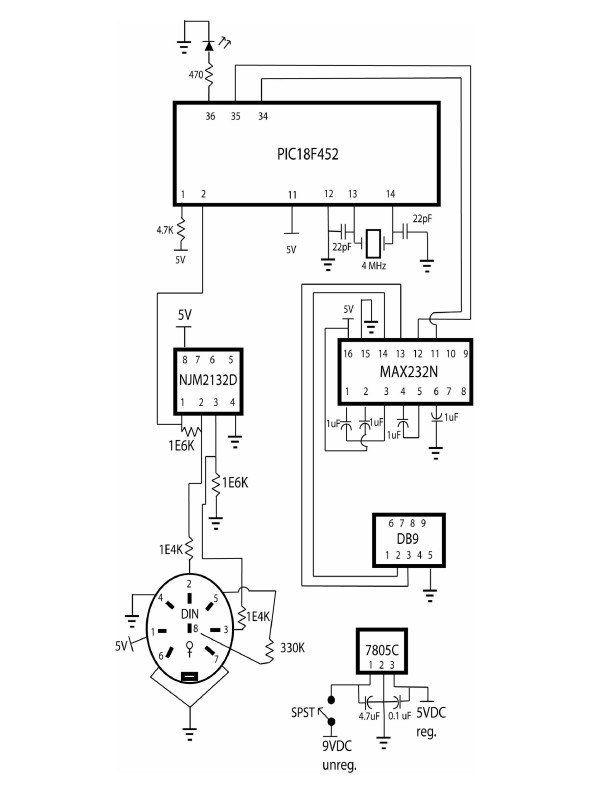
Schematic of petal breakstrength electronic circuit. Pins on the PIC18F452 that were unused are not drawn. All resistors are rated at 0.25 watts and electrolytic capacitors are rated for 50 volts.

The assembled circuit is shown in Figure 1F and a schematic of the circuit in Figure 2. The MLT050 force transducer uses a Wheatstone bridge circuit to convert applied force to a voltage potential. A voltage potential is applied to one diagonal of the bridge for device excitation and the potential difference across the other diagonal is the sensor's measured output. When the sensor cantilever is depressed, the resistance of the bridge's strain-sensitive resistors is altered, changing the output potential. A single supply operational amplifier configured as a differential amplifier is used to amplify the signal from the sensor. An embedded microcontroller, the Microchip PIC18F452, was used to perform analog to digital signal (ADC) conversion and serial communication to output the data to a computer. The 18F452 has a 10 bit ADC, so with 5 V reference voltage, the ADC has a resolution of approximately 5 mV. The MAX232 converts the transistor-transistor level (TTL) signals from the microcontroller to RS232 levels needed for serial communication with the personal computer. The 7805C provides a 5 VDC regulated supply for sensor excitation, ADC reference voltage, and power for the other chips. Although we used a 9 V wall transformer for power, power could alternatively be supplied by an 9 V battery.

**Figure 3:**
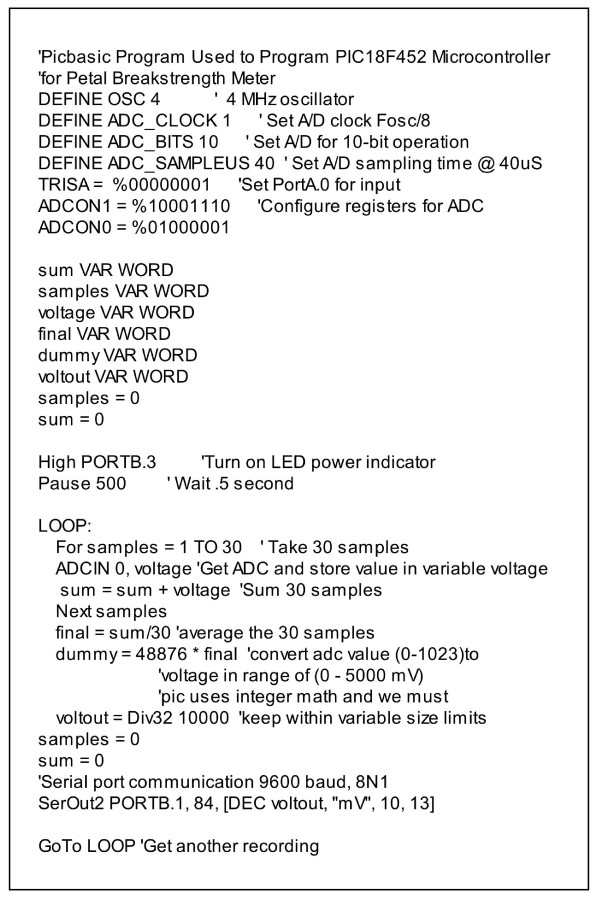
Picbasic program used to program PIC18F452 microcontroller in petal breakstrength meter. The code documentation comments are preceeded by apostrophes.

The microcontroller was programmed with a program written in Picbasic (Figure 3), compiled to assembly using the Picbasic Pro Compiler (microEngineering Labs, Inc., Colorado Springs, CO), and used to program the PIC18F452 using a PIC programmer (K128 USB Flash Pic programmer, KitsRUs, ). The assembly version of the program is available as supplementary data to this publication. Alternatively, the programmed microcontroller can be requested from the authors for a nominal fee to cover the cost of the part and shipping.

### Improving sensitivity of MLT050 sensor to small forces

The MLT050 force transducer has a range of 0 – 50 grams and outputs a voltage signal that varies linearly with the applied force. However, since the voltage output of the sensor is so small (at 5 V device excitation, 50 g of applied force causes an output of less than 100 mV), directly measuring the voltage output of the sensor is not ideal. Direct measurement of the output of the sensor would prevent detecting subtle differences in petal breakstrength that may exist between abscission defect mutants.

Arabidopsis petal breakstrength requires several grams or less of force equivalents (Butenko et al., 2003). To improve the sensitivity of the meter to this range of measurement, we introduced two design features. First, we used mechanical leverage to improve sensitivity. A 5 cm long aluminum strip in which small holes were drilled at both ends was employed as a lever (Fig. 1B). The strip was fastened to the sensor with a screw and nut. Second, we linearly amplified the voltage signal output from the sensor by a factor of 100 using an operational amplifier (Fig. 2). These elements enabled the meter to have higher sensitivity to small forces and higher accuracy in the force range of interest.

### Sensor attachment to Arabidopsis petals via petal gripper

Due to the miniscule size of Arabidopsis flowers, physically attaching the sensor to a flower petal was a challenging design issue. We initially tried micro-alligator clips, but found that they were too large and cumbersome. Instead, we used a clip that is designed for attaching to the legs of a surface mount device (SMD) integrated chip, called an 'SMD grabber.' The tips of the off-the-shelf SMD grabber were found to be too sharp and fine. As a consequence, during the assay, the flower petal would tear rather than be pulled out of the flower. We superglued ~1 mm long pieces of insulation from 20 gauge wire over the tips of the SMD grabber (Fig. 1C–E). This modification spreads out the pressure of the SMD grabber tips to a wider surface of the flower petal, and solved the petal tearing problem. The petal gripper is coupled to the sensor via a short piece of 20 gauge braided copper wire passed through the SMD grabber plunger and the drilled hole on the distal end of the 5 cm aluminum strip (Fig. 1B).

### Device validation: linearity of response

**Figure 4:**
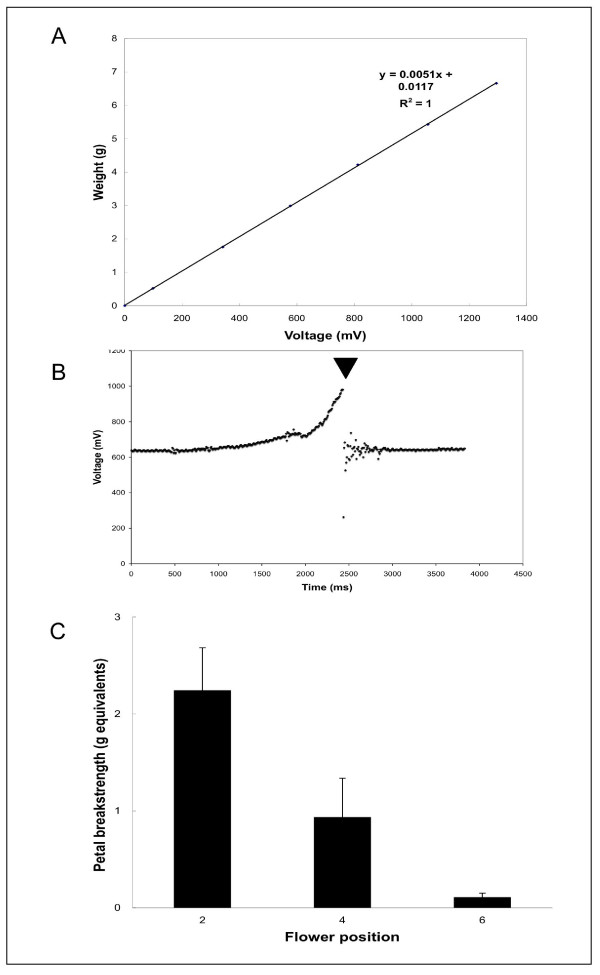
Validation of the petal breakstrength meter. (**A**) Linearity of standard curve of voltage observed as a function of weight. The fitted linear equation and the correlation coefficient are shown in the upper right hand corner. (**B**) Data from a typical petal breakstrength assay recording. A wildtype Arabidopsis stage 13 [12] flower-attached petal was gripped by the breakstrength meter petal gripper and then recording was begun. The inflorescence was pulled down (ascending voltage) until the petal was pulled out of the flower (indicated by the triangle). Following this event, the voltage fluctuates briefly due to the damped harmonic motion of the lever following petal pulling. (**C**) Petal breakstrength assay results. Two petals per flower were assayed from 15 wildtype *Arabidopsis thaliana *Columbia ecotype plants at the indicated flower positions (n = 30 petals per flower position). Bars represent mean ± standard deviation. Flower 1 is defined as the youngest flower on the inflorescence in which the flower petals are visible.

Although the voltage should vary linearly with respect to weight, it was important to test this directly. For example, it was formally possible that the circuitry used to amplify the signal could cause distortion if too much gain was used, resulting in a deviation from linearity. To test the linearity of the device, we established a standard curve (Figure 4A). The linearity of the petal breakstrength meter was excellent, with a correlation coefficient of 1 indicating that we did not introduce any distortion in the force range of interest. Based on the 10 bit ADC resolution giving a voltage resolution of 4.8876 mV (5000 mV/1023), and using the standard curve equation, the petal breakstrength meter has a force resolution of approximately 25 mg.

### Device validation in the petal breakstrength assay

To show the high resolution of data acqusition and visualize the data from an assay recording, we plotted the voltage measurements from a typical petal breakstrength trial (Figure 4B). The rate of data acquisition is greater than 100 measurements per second. During the petal breakstrength assay, the voltage is observed to rise as the petal is pulled until the moment the petal detaches from the receptacle. Following the petal pulling, the voltage briefly fluctuates as the lever vibrates with a harmonic motion before settling down to basal levels.

We tested our petal breakstrength meter in the petal breakstrength assay to see if the values we measured were comparable to those reported in the literature (Fig. 4C). For example, our measured average value of 2.24 grams for position two flower petals is close to the range of force (~1.75 g, ~2.5 g and ~1.75 g respectively) reported by Fernandez et al. (2000), Butenko et al. (2003), and Patterson and Bleecker (2004) in wildtype petal breakstrength assays. Therefore, we concluded that our device for petal breakstrength assay performed similarly to the preexisting device.

**Figure 5:**
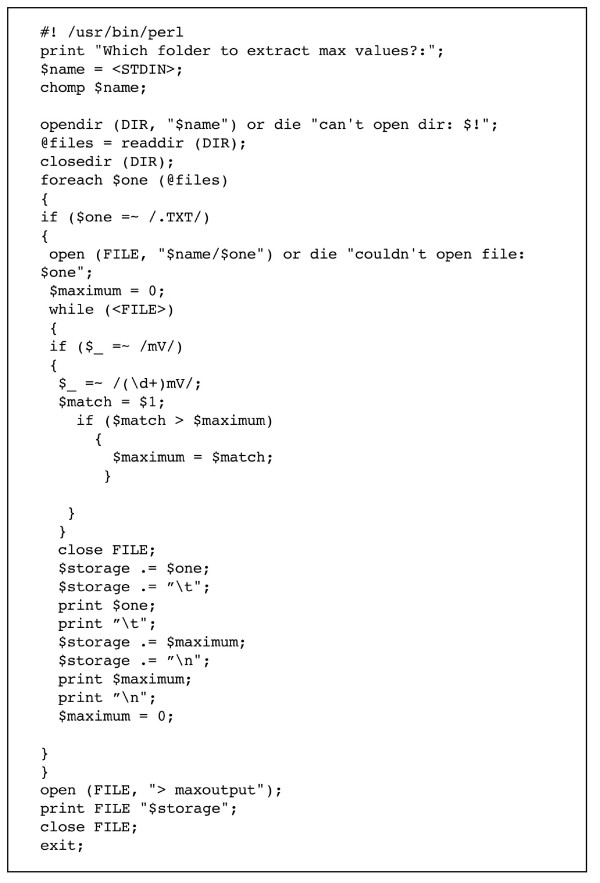
Perl script findforces.pl used to facilitate extraction of petal breakstrength values from a folder of petal breakstrength assay text capture files.

To avoid visually searching for the captured recording files for the force that causes the petal to detach, we developed a simple Perl script called findforces.pl which reports the maximum measured value (Fig. 5). This enables petal breakstrengths to be formatted into a single tab-delimited file for import into Excel or other software for statistical analysis.

## Discussion

We have demonstrated the design and use of a petal breakstrength meter for Arabidopsis abscission studies. The detailed description of this device should enable other researchers to build the meter so that measuring petal abscission breakstrength will become a routine assay in studying new mutants. Excluding the personal computer, the cost of the parts used in construction (Table 1), is about $300 with the sensor being the most expensive single part. Assembling the device requires a soldering iron and about two hours of time. Therefore, the cost and time required for assembly of the petal breakstrength meter are not prohibitively high.

We have considered several future technical improvements of the device. For example, using an external 16 bit ADC instead of the onboard 10 bit ADC on the PIC18F452 would enable higher resolution measurements. In terms of speed of data acquisition, the serial transmission of data is the rate-limiting step. Two methods could be employed to improve this speed. First, a higher speed, e.g. 20 MHz crystal could be used for clocking the PIC, so that a higher rate of serial communication could be used reliably. Alternatively, the device could be modified to send the voltage measurement as two bytes of binary data, rather than a multibyte string of ASCII characters representing the decimal version of the data. This change would be rather easy to include as it would only involve modifying the firmware of the chip, rather than modifying the circuit hardware.

In principle, the petal breakstrength meter we designed could be easily adapted for measuring the physical integrity of many other materials (biological or non-biological). The main limitation is the ability to hook the item of interest to the sensor. Another consideration in the context of modifying the circuit to other applications is the range of sensitivity of the device. The amount of voltage amplification can be easily modified by replacing the 10 K ohm resistors with matched resistors of other values. Another way of modifying the sensitivity is to simply change the length of the lever attached to the sensor's built-in cantilever.

**Table 1:** Parts and Suppliers for Construction of Petal Breakstrength Meter

(1) MLT050/D Force Transducer (ADInstruments, )
(1) NJM2132D (Mouser, , p\n 513-NJM2132D)
(1) SMD grabber clip (Mouser, p\n 565-5243-0)
(2) 16 pin DIP sockets (Mouser, p\n 575-199316)
(1) PIC18F452-I/P (Glitchbuster, p\n PIC18F452-I/P).
(1) 7805 voltage regulator (Glitchbuster, p\n 7805)
(1) MAX232N (Glitchbuster, p\n MAX232N)
(1) 4 MHz crystal (Glitchbuster, p\n XT-4)
(2) 22 pf capacitors (Glitchbuster, included with crystal)
(1) 4.7K ohm 0.25W resistor (Glitchbuster, p\n R4.7k)
(1) 470 ohm resistor 0.25W (Glitchbuster, p\n R470)
(5)10K ohm resistors (Glitchbuster, p\n R10K)
(2) 1.0 Mohm W resistors (Glitchbuster, p\n R1.0M)
(3) 100 Kohm W resistors (Glitchbuster, p\n R100k)
(4) 1.0 uF electrolytic capacitors (Glitchbuster, p\n 1R50)
(1) 4.7 uF electrolytic capacitor (Glitchbuster, p\n 4.7R50)
(1) 0.1 uF capacitor (Glitchbuster, p\n .1UF-MONO)
(1) Red LED (Glitchbuster, p\n LED-R)
(1) Serial cable (Glitchbuster, p\n DB9-6 M-F)
(1) STD DIN-8 female connector (MPJA, , p\n 5616 PL)
(1) D subminiature 9 female right angle connector (MPJA, p\n 4840 PL)
(1) SPST switch (MPJA, p\n 5002 SW)
(1) ABS project enclosure (MPJA, p\n 15521 BX)
(1) Power supply 9V 1A or similar (MPJA, p\n12254 PD)
(1) perforated board (Radio Shack, p\n 276-147)
(1) 40 pin socket (Radio Shack, , p\n 276-1996)

## Conclusion

We developed and tested a new petal breakstrength meter that provides a significant advance in the petal breakstrength assay used in Arabidopsis abscission studies. This should enable researchers to make this assay a routine part of their characterization of abscission mutants. In addition, the device may find unanticipated applications in scientific assays involving measurements of the physical integrity of other materials.

## Methods

### Device assembly

The electronic parts were obtained from the suppliers listed in Table 1. This list is given for convenience and should not be interpreted to read that the parts can only be obtained from the stated sources (with the exception of the force transducer). Multiple alternate vendors of the parts can be found by searching the internet.

In the event that the PIC18F452 is unavailable, the PIC18F4520 may be used as an alternative without modifying the circuit or Picbasic source code. The programmed device may be obtained by request from the corresponding author. Alternatively, the programmed PIC may be purchased from a commercial PIC programming service provider (e.g. ).

The circuit was first assembled on a solderless breadboard. After confirming that everything was working correctly, the circuit was assembled on a perforated prototyping board, using a soldering iron and a 20 gauge wire for jumper connections. The lever was a 5 cm long × 0.5 cm wide × 0.1 cm thick piece of aluminum that was cut from a piece of aluminum with tin snips, hammered flat, and 1 mm diameter holes were drilled about 3 mm from each end.

### Petal breakstrength assay

A standard curve (voltage as a function of weight) was generated by sequentially measuring the voltage generated with several objects (paper clips) of known weight suspended from the petal gripper. The voltage measured without any extra weights attached was subtracted from voltages of all weights, to correct for the weight contribution of the petal gripper and lever. The data were fitted to a linear curve using Microsoft Excel to obtain the y = mx + b equation. When an unknown (i.e. a petal pull) is measured, the observed voltage (after performing the aforementioned voltage subtraction from the weight of the petal gripper and lever alone) was used to calculate the gram equivalents of force using the standard curve. The petal pulling was performed manually. The maximum voltage value from the acquisition during petal pulling was used to calculate the petal breakstrength. We used 3.5 week old *Arabidopsis thaliana *Columbia ecotype plants that were grown at a temperature of 22°C temp with 16 h 100 μmolm^-2^sec^-1 ^light/8 h dark. The petal could be seen in the jaws of the petal gripper following pulling, as a visual confirmation that the petal did not slip out or tear during the assay (Fig. 1E).

## Competing interests

The author(s) declare that they have no competing interests.

## Authors' contributions

KL designed and built the breakstrength meter, SKC performed the breakstrength assays, and JCW contributed to the concept of an inexpensive, accessible breakstrength meter. All authors helped draft the manuscript, have read and approved the final manuscript.
